# The Cancer Immunotherapy Thromboembolism Assessment: A Novel Score for Predicting Thromboembolic Events in Melanoma Patients Treated With Immune Checkpoint Inhibition

**DOI:** 10.1002/ijc.70523

**Published:** 2026-04-27

**Authors:** Tim Zell, Julian Kött, Noah Zimmermann, Greta Ancker, Alexander T. Bauer, Daniel J. Smit, Glenn Geidel, Julian C. Gerwers, Thomas Renné, Sebastian A. Wohlfeil, Jochen Utikal, Stefan W. Schneider, Christoffer Gebhardt

**Affiliations:** ^1^ Department of Dermatology and Venereology University Medical Center Hamburg‐Eppendorf Hamburg Germany; ^2^ Fleur Hiege Center for Skin Cancer Research University Medical Center Hamburg‐Eppendorf Hamburg Germany; ^3^ Institute of Tumor Biology University Medical Center Hamburg‐Eppendorf Hamburg Germany; ^4^ Institute of Clinical Chemistry and Laboratory Medicine University Medical Center Hamburg‐Eppendorf Hamburg Germany; ^5^ Department of Cardiology University Heart and Vascular Center, University Medical Center Hamburg‐Eppendorf Hamburg Germany; ^6^ Irish Centre for Vascular Biology, School of Pharmacy and Biomolecular Sciences Royal College of Surgeons in Ireland Dublin Ireland; ^7^ Skin Cancer Unit German Cancer Research Center (DKFZ) Heidelberg Germany; ^8^ Department of Dermatology, Venereology and Allergology University Medical Center Mannheim, Ruprecht‐Karl University of Heidelberg Mannheim Germany; ^9^ DKFZ Hector Cancer Institute at the University Medical Center Mannheim Mannheim Germany

**Keywords:** cancer associated thromboembolism, immune checkpoint inhibition, melanoma, risk assessment model, thromboembolic events

## Abstract

Venous and arterial thromboembolic events (TEEs) represent a substantial threat for melanoma patients treated with immune checkpoint inhibition (ICI) and have a significant impact on quality of life, therapy outcome, and survival. Existing risk assessment models for predicting TEE risk have been developed for other patient collectives and show poor performance in melanoma patients treated with ICI. In this cohort analysis, 358 AJCC stage III/IV melanoma patients treated with ICI between April 2013 and July 2024 at the University Skin Cancer Center Hamburg and the University Medical Center Mannheim were included. TEEs were recorded and classified as thrombosis including vein thrombosis, pulmonary embolism, stroke, or transient ischemic attack. Clinical and laboratory data were determined before the start and prospectively during the treatment. We identified elevated serum baseline D‐Dimer (*p* = 0.0098) and elevated C‐reactive protein (*p* = 0.0042) concentrations and measurable tumor burden (*p* = 0.0039) as main risk factors for the occurrence of TEE. For the final model, points were assigned for the Cancer Immunotherapy Thromboembolism Assessment (CITA) according to the impact of those variables using multiple logistic regression. The score was calculated for each patient. For the high‐risk group, the negative predictive value (NPV) was 97.2%; sensitivity and specificity were 83.3% and 62%, respectively. The CITA risk score provides a simple and easily calculated risk assessment tool for stratifying melanoma patients based on their risk for TEE after ICI initiation, but prospective validation is needed before clinical use can be recommended.

## Abstract (highlights)

What's new?

Although immune checkpoint inhibitors (ICI) have transformed metastatic melanoma treatment, thromboembolic events (TEEs) remain a serious complication. Moreover, existing risk assessment models were developed prior to ICI use and lack clinical validation. Here, the authors investigated various predictors to develop a risk model tailored specifically for ICI‐treated stage III/IV melanoma patients. Elevated D‐dimer and C‐reactive protein levels and measurable disease were identified as key risk factors. These measures were integrated into a Cancer Immunotherapy Thromboembolism Assessment (CITA) score, which demonstrated high sensitivity and negative predictive value. The findings highlight CITA's potential as an ICI‐specific tool for individualized TEE risk stratification.

## Graphical abstract

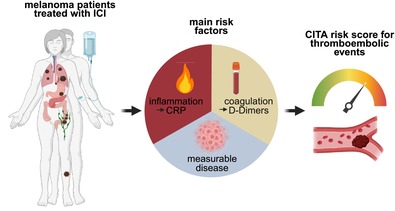

**Abbreviations**
ADOReg: German Skin Cancer Registry
AJCC: American Joint Committee on Cancer
BMI: body mass index
CAT: cancer associated thromboembolism
CATScore: Cancer Associated Thrombosis Score
CITA: Cancer Immunotherapy Thromboembolism Assessment
CRP: C‐reactive protein
CTLA‐4: Cytotoxic T‐lymphocyte associated protein 4
ECOG: Eastern Cooperative Oncology Group
Hb: hemoglobin
ICI: immune checkpoint inhibition/inhibitors
LDH: lactate dehydrogenase
NPV: negative predictive value
OS: overall survival
PD‐1: programmed cell death protein 1
PFS: progression‐free survival
PPV: positive predictive value
RAM: risk assessment model
ROC: receiver operating characteristic
TEEs: thromboembolic events
TF: tissue factor
VEGF: vascular endothelial growth factor
vWF: von Willebrand Factor

## 1 Background

Immune checkpoint inhibition (ICI) has emerged as the leading strategy for the treatment of metastatic melanoma and has begun augmenting and replacing chemotherapeutic regimens in a variety of other tumors [1, 2, 3, 4]. This immunologic approach to cancer therapy is usually accompanied by fewer side effects than traditional chemotherapy, which targets all fast‐growing cells in the patient's body [5]. Still, a variety of cancer‐ and treatment‐related adverse events occur in patients treated with immune checkpoint inhibitors [6]. Among those adverse events in cancer patients, venous and arterial thromboembolic events (TEEs) represent a substantial threat that can have a significant impact on quality of life, therapy outcome, and survival [7, 8, 9]. Because the highest incidence of TEE is observed in patients with gastrointestinal malignancies treated with chemotherapy, studies concerning TEE in cancer usually focus on this patient collective [10, 11]. But since the emergence of ICI as a new treatment option for patients, research has begun to shed light on the importance of TEE in those patients [12, 13]. A retrospective cohort study conducted by Sussman et al. [14] reported that one out of five melanoma patients treated with ICI experiences a TEE throughout therapy and that the occurrence of TEE adversely correlates with overall survival (OS).

Various studies have explored the mechanisms by which cancer cells interact with the surrounding microenvironment to promote a procoagulatory environment, which includes the expression of tissue factor (TF), matrix metalloproteinase‐1, and Vascular Endothelial Growth Factor (VEGF) [15, 16, 17, 18, 19]. In melanoma, Bauer et al. [20], have shown that malignant cells express TF and secrete VEGF‐A to interact with endothelial cells in distant organs such as the liver, the brain and the lung. This process promotes the luminal release of the procoagulatory protein von Willebrand Factor (vWF) followed by platelet aggregation and thrombotic vessel occlusions.

The induction of a procoagulatory environment by cancer cells promotes disease progression by facilitating local invasion, distant metastasis, immune evasion, and resistance to anti‐cancer therapies [20, 21, 22, 23]. To counteract this fatal interplay between coagulation and cancer, several studies suggest the usage of anticoagulant or antithrombotic medication, like the low‐molecular weight heparin tinzaparin and Factor Xa Inhibitors, which were shown to prevent those interactions in preclinical models [20, 24, 25]. Graf et al. [24], even demonstrated that Factor Xa inhibition using Rivaroxaban (Bayer AG) acts synergistically with ICI to improve anti‐tumor immunity [24].

The available preclinical data on the role of coagulation in cancer, and the high incidence of TEEs, suggest a possible benefit of using anticoagulant or antithrombotic medication to counteract the interplay and prevent potentially fatal events. However, clinical data on the widespread use of anticoagulation in cancer patients treated with ICI has been inconsistent [26, 27]. A recent evaluation of the German Skin Cancer Registry ADOReg showed that in a cohort of 2419 melanoma patients treated with ICI, concomitant oral anticoagulation was significantly associated with an improved overall and progression‐free survival (PFS), further suggesting a potential synergistic effect [28].

However, a widespread use of concomitant anticoagulation in cancer patients cannot be justified based on the available data, and the presence of certain risk factors for cancer‐associated TEEs suggests that coagulation activation and TEE risk have to be evaluated in each patient individually in order to avoid unnecessary exposure to bleeding risk associated with anticoagulation [11, 29]. For that reason, several score systems have been developed, most notably by Khorana et al. [30], and the COMPASS‐CAT investigators, using variables such as hemoglobin, body mass index (BMI), leukocyte count, cancer type, and medical history [30, 31] These risk assessment models (RAMs) are of tremendous help in clinically identifying patients with an increased risk for TEE.

However, they have been developed before the era of ICI and lack clinical validation in this treatment cohort. For melanoma patients treated with ICI, there is no validated clinical model for predicting TEE risk to this day. In this study, we aim to develop a RAM to stratify melanoma patients under ICI based on their risk for TEEs.

## 2 Methods

### 2.1 Patient Cohort

The study population was comprised of patients treated at the Skin Cancer Centers of the University Medical Centers of Hamburg and Mannheim. All patients were required to have a histologically confirmed diagnosis of completely resected or advanced, metastatic melanoma (AJCC stage IIB‐IV), and to have received at least one cycle (one infusion) of any ICI. Patients were enrolled and followed up between 2013 and 2025. TEEs were recorded as diagnosed by the treating physicians and classified as thrombosis (e.g., deep vein thrombosis), stroke, pulmonary embolism or transient ischemic attack. Clinical and laboratory data, including differential blood count, D‐Dimers, C‐reactive protein (CRP), Lactate dehydrogenase (LDH) and Hemoglobin (Hb), were determined before the start and prospectively during the treatment before administration of treatment using Siemens Atellica Solution analyzers. To be included in the derivation of the risk score, patients were required to have sufficient clinical and laboratory data available before the first cycle of therapy. All treatment decisions were made independently from this study.

### 2.2 Statistical Analysis

Clinical variables previously linked to TEEs, along with potential risk factors, were considered for the derivation of the model. These included patient demographics such as age and sex, Eastern Cooperative Oncology Group (ECOG) performance status, AJCC stage, therapy regimen, obesity (defined as a BMI > 35 kg/m^2^), concomitant anticoagulation, history of TEE, and Khorana score. Only variables that could be assessed prospectively before the first cycle of ICI were included, as the objective of the score is to identify high‐risk individuals before treatment start. Baseline laboratory values were first examined as continuous variables, and then a cutoff value was determined using the receiver operating characteristic (ROC) and Youden Index method to ensure clinical usability of the score. Variables were evaluated in univariate analysis using the Chi‐squared test.

For model derivation, a multivariate logistic regression was performed. To ensure model stability, the final model was required to include a maximum of three variables. The final risk score was developed based on the final odds ratio for each variable. Discrimination was quantified using conventional metrics including sensitivity, specificity, and predictive values. To provide an overall measure of discrimination, the area under the ROC curve was calculated. Predictive value over time was determined using a Kaplan–Meier time to event analysis. Statistical analysis was performed using Graphpad Prism version 9.5.1 and R version 4.4.3. Packages used include: readxl, survival, survminer, dplyr, broom, ROCit, gridExtra (versions May 2025). A *p*‐value ≤ 0.05 was considered statistically significant.

## 3 Results

### 3.1 Cohort Characteristics

The present study cohort comprised 358 melanoma patients treated with ICI. A total of 290 patients received treatment at the University Skin Cancer Center in Hamburg, with 68 of these patients treated at the Skin Cancer Center of the University Medical Center Mannheim. During the study, 24 patients (6.7%) exhibited the occurrence of a TEE following the initiation of therapy. The most prevalent diagnoses were pulmonary embolism (*n* = 8), thrombosis (*n* = 8; e.g., deep vein, portal vein or other), or stroke or transient ischemic attack (*n* = 6). The median follow‐up period was 20 months. The characteristics of the cohort are delineated in Table 1 and in Tables S1 and S2.

**TABLE 1:** Cohort characteristics.

Variable	*N*	(%)
Age group		
≥ 65	196	54.75
< 65	162	45.25
Sex		
Male	214	59.78
Female	144	40.22
ECOG		
0	282	78.77
≥ 1	76	21.23
AJCC Stage		
III	183	51.12
IV	175	48.88
Measurable disease		
No/post full resection	152	42.46
Yes	206	57.54

Abbreviation: ECOG, Eastern Cooperative Oncology Group Performance status.

### 3.2 Derivation of a Risk Model for Thromboembolic Events

Initially, a univariate analysis was conducted to assess the potential risk factors. The following variables were found to be significantly associated with the occurrence of TEEs following therapy initiation: Anti‐PD‐1 monotherapy (*p* = 0.0335, Ipilimumab+Nivolumab vs. Nivolumab or Pembrolizumab), serum D‐dimer concentrations ≥ 0.6 mg/L, serum CRP concentrations ≥ 10 mg/L, and the presence of measurable disease before the first ICI cycle (*p* = 0.0335, not fully resected tumor or presence of distant metastasis). The findings of the study showed that age, BMI, ECOG performance status, and Khorana score were not significantly associated with the occurrence of TEE, neither as continuous nor as dichotomized variables (Table 2). Importantly, none of the dichotomized parameters included in the Khorana risk score for venous thromboembolism in cancer patients showed a statistically significant association with TEEs in univariate analysis. These parameters comprise leukocyte count, platelet count, hemoglobin level or the use of red blood cell growth factors, and BMI (Table S3).

**TABLE 2:** Univariate analysis of dichotomized risk factors for the development of thromboembolic events (TEE) after immune checkpoint inhibition (ICI) initiation.

Variable	Odds ratio	*p*
Combination ICI (Nivolumab+Ipilimumab)	2.433	**0.0335**
ECOG	1.956	0.1921
Measurable disease	4.435	**0.0039**
BMI > 35 kg/m^2^	0.9307	0.6760
Khorana score ≥ 1	1.315	0.5415
D‐Dimers ≥ 0.6 mg/L	3.755	**0.0098**
CRP ≥ 10 mg/L	3.25	**0.0042**
Previous TEE	0.502	0.2830

*Note:* significant *p*‐values (< 0.05) are highlighted in bold.

In a stepwise multiple logistic regression model, the three most significantly associated variables were selected and assigned points according to their individual impact. For the final CITA risk score, measurable disease and elevated baseline D‐dimers ≥ 0.6 mg/L were assigned a point each, while baseline CRP ≥ 10 was counted as a point (see Table 3). The final CITA score was calculated for each patient, and patients were stratified into three risk groups according to their individual TEE risk: according to the criteria outlined in Table 3, the risk level is categorized as follows: low risk, assigned a value of 0 points; intermediate risk, assigned a value of 1 point; and high risk, assigned a value of 2 or more points.

**TABLE 3:** Development of the risk assessment model using multiple logistic regression.

Risk factor	Odds ratio	95% CI	Risk points
D‐Dimers ≥ 0.6 mg/L	2.54	0.92 to 8.16	1
CRP ≥ 10 mg/L	1.85	0.74 to 4.64	1
Measurable disease	2.74	0.95 to 9.90	1

*Note:* Low risk = 0 points, intermediate risk = 1 point, high risk = 2 or more points.

### 3.3 Characteristics and Validation of the Risk Assessment Model

**FIGURE 1:**
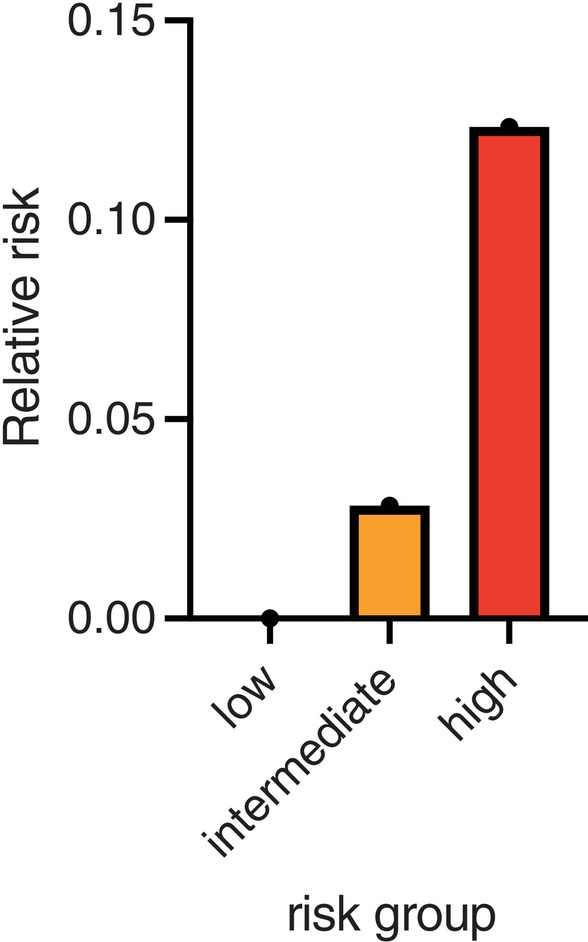
Relative risk for a thrombotic event under ICI. Relative risk for developing any kind of thromboembolic event (TEE) after immune checkpoint inhibition (ICI) initiation, stratified according to three risk groups: Low (0 points), intermediate (1 points), and high risk (≥ 2 points).

**FIGURE 2:**
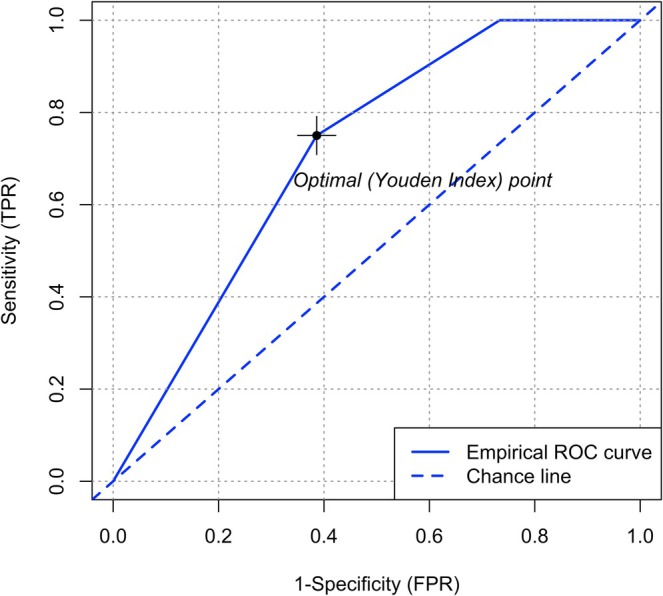
Receiver‐operating characteristic analysis of the risk assessment model. Area under the curve = 0.715. The optimal cut off (≥ 2 points) was calculated using the Youden Index method.

**FIGURE 3:**
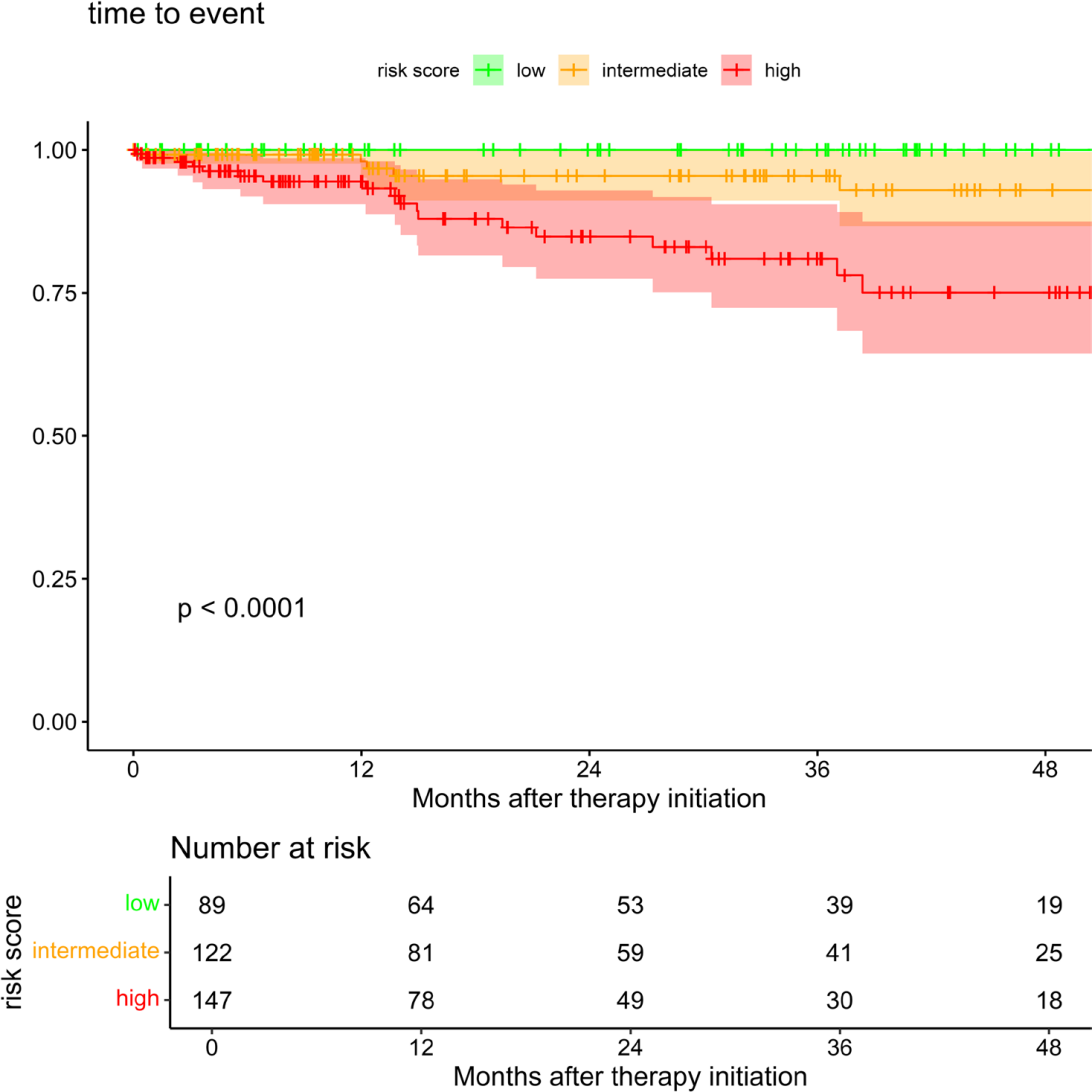
Time to thromboembolic event analysis. Kaplan–Meier time to thromboembolic event (TEE) analysis of the three risk groups according to the Cancer Immunotherapy Thromboembolism Assessment (CITA) risk assessment model (RAM): Low‐ (0 points), intermediate (1 point), high‐risk (≥ 2 points). Statistical significance was assessed by the log‐rank test.

In the low‐risk cohort (*n* = 89), no patient exhibited the development of a TEE during the entire follow‐up period. In the intermediate group, 6 out of 122 patients (4.9%) were affected by any kind of TEE. In the high‐risk cohort, 18 out of 147 patients developed a TEE after therapy initiation (12.2%) (Figure 1). When the high‐risk cutoff value (≥ 2 points) was evaluated against the low/intermediate risk group, the test demonstrated a negative predictive value (NPV) of 97.2% and a positive predictive value (PPV) of 12.2% for predicting TEE following therapy initiation. The sensitivity and specificity levels were recorded as 83.3% and 62%, respectively. The area under the ROC curve was determined to be 0.715, as illustrated in Figure 2. The median time to occurrence of the event was 13.7 months. The discrepancy in TEE incidence persisted as statistically significant over the course of the study (*p* < 0.0001, Figure 3). In the initial year of the study, two patients in the intermediate group experienced a TEE, while seven events were documented in the high‐risk group.

## 4 Discussion

Using data from an observational bicentre study, we derived a RAM to predict the occurrence of TEEs in melanoma patients after initiation of ICI treatment, using data available before the start of the first cycle. The score was especially effective in identifying a low‐risk cohort, in which no events occurred during therapy, enabling a focus of potential preventive measures on a high‐risk population. The parameters of the CITA risk score model include the presence of measurable disease, CRP and D‐Dimer concentrations in the patient serum. These three variables comprise important influencing factors of cancer‐associated thromboembolism (CAT). Cancer cells themselves, as well as an inflammatory anti‐cancer response, indicated by elevated CRP concentrations, can activate coagulation and therefore facilitate clot formation [32, 33]. In addition, an elevation of D‐Dimers, routinely used in everyday clinical practice to rule out a TEE as a fibrin degradation product resulting from ordered breakdown of thrombi by the fibrinolytic system, may indicate a higher baseline activity of the coagulation system in the patient [34].

Previous RAMs such as the Khorana score were primarily developed for other cancer types and for patients receiving chemotherapy and may assign risk points for cancer types other than melanoma [30, 31]. The Khorana score, which is the most commonly used RAM in clinical practice, did not correlate with the development of TEE after therapy initiation (Table 2). The Khorana score, which remains the most commonly used RAM in clinical practice, did not correlate with the development of TEE after therapy initiation in our cohort (Table 2). Notably, the Khorana score includes cancer type as a variable and assigns points for various cancer types, not including melanoma. Moreover, none of its remaining components (BMI, leukocyte count, hemoglobin level, platelet count) demonstrated a significant association with TEE in our univariate analyses (Table S3). This likely reflects limited transportability of the Khorana score to melanoma patients treated with ICI, a population that differs substantially from the original derivation cohort in both tumor biology and treatment context. Traditional CAT risk factors incorporated in the Khorana score were less prevalent in our cohort. For example, only 6.1% of patients had an elevated BMI, and leukocytosis was observed in less than half of the patients compared to the reported observations in the original cohort used to establish the Khorana score (> 12%) [30]. Beyond differences in baseline characteristics, accumulating evidence suggests that ICI therapy may promote TEEs via immune‐mediated mechanisms [35, 36]. These pathways are not reflected in the clinical and laboratory variables included in the Khorana score, which was developed in patients receiving cytotoxic chemotherapy. Consequently, the biological drivers of TEEs in melanoma patients treated with ICI may differ from those captured by the Khorana score. In contrast, our RAM was calculated specifically in melanoma patients receiving ICI therapy, thereby capturing risk factors and clinical patterns unique to this population.

This observation is in accordance with a recent publication by Vladic et al. [37], which showed that most previously developed RAMs show poor performance in contemporary cohorts of cancer patients including those treated with ICI. In their study, the CATScore, which includes D‐Dimer concentration and cancer site (with no specific points assigned for melanoma), performed best, confirming the benefit of measuring D‐Dimers as a marker for coagulation activity in cancer patients [37]. In strong accordance, Desch et al. [38], could already show that elevated concentrations of D‐Dimers at baseline are significantly associated with decreased OS and PFS in melanoma patients. The prognostic value of D‐Dimers concerning OS, PFS, and TEE risk suggests an urgent need for further investigation of their role as a biomarker. Notably, D‐Dimers are measured routinely in everyday clinical practice to rule out a TEE and should therefore be easily available at any laboratory.

When we compared the measurement of D‐Dimers as a continuous variable, as it is used in the CATScore, to our RAM, the predictive value of D‐Dimers alone was less than that of our score, which includes three dichotomized variables. The area under the ROC curve was 0.66 for D‐Dimers and 0.715 for our RAM, where patients can be stratified by simple addition of points for the three variables. However, the CITA risk score, as proposed in this study, urgently requires prospective validation before any conclusions can be drawn from this score in clinical routine. The main strength of this score is its high NPV, and usage of this score would be especially valuable in identifying patients at low risk for TEE. Therefore, the CITA score could help in effectively allocating diagnostic and preventive resources. Further research could explore the predictive value of other markers of the coagulation cascade, such as ADAMTS13 (a disintegrin and metalloproteinase with a thrombospondintype 1 motif, member 13) and vWF, which are not yet available for routine measurement in the clinic, but may provide predictive value for therapy outcome and possibly TEE in cancer patients [39, 40, 41].

Furthermore, the goal of this study was to develop a score to classify patients at baseline based on their TEE risk after therapy initiation. Investigating the dynamic changes of these variables during therapy could provide an effective tool to constantly monitor and re‐evaluate disease dynamics and TEE risk in cancer patients [42].

## 5 Conclusion

Immune checkpoint inhibition (ICI) has emerged as the leading strategy for the treatment of metastatic melanoma, and TEEs represent a substantial threat that can have a significant impact on quality of life, therapy outcome, and survival. The CITA score provides a simple risk stratification based on three parameters: the presence of measurable disease as well as baseline D‐Dimer (≥ 0.6 mg/L) and CRP (≥ 10 mg/L) concentration in the patient serum. A prospective study is needed to validate this score for clinical use.

## Author Contributions

**Tim Zell:** conceptualization, investigation, writing – original draft, writing – review and editing, visualization, methodology, validation, software, formal analysis, project administration, data curation, resources. **Julian Kött:** conceptualization, investigation, writing – review and editing, methodology, visualization, writing – original draft, software, formal analysis, project administration, data curation, resources. **Noah Zimmermann:** investigation, writing – review and editing, methodology, formal analysis, resources, validation. **Greta Ancker:** investigation, writing – review and editing, methodology, validation, formal analysis, data curation. **Alexander T. Bauer:** writing – review and editing, methodology, investigation, data curation, supervision, resources. **Daniel J. Smit:** data curation, supervision, resources, writing – review and editing. **Glenn Geidel:** writing – review and editing, data curation, resources, supervision. **Julian C. Gerwers:** data curation, supervision, resources, writing – review and editing. **Thomas Renné:** writing – review and editing, data curation, supervision, resources, methodology. **Sebastian A. Wohlfeil:** writing – review and editing, data curation, supervision, resources. **Jochen Utikal:** data curation, supervision, resources, writing – review and editing. **Stefan W. Schneider:** writing – review and editing, data curation, supervision, resources, project administration. **Christoffer Gebhardt:** project administration, data curation, supervision, resources, conceptualization, investigation, methodology, validation, writing – review and editing, formal analysis.

## Funding

T.Z. was supported by the Else Kröner‐Fresenius‐Stiftung iPRIME‐CS Scholarship (2021_EKFK.15), UKE, Hamburg.

## Ethics Statement

This study was conducted in accordance with the Declaration of Helsinki and approved by the Ethics Committee of the Hamburger Ärztekammer (PV5392). All patients provided written informed consent.

## Conflicts of Interest

J.K. has received honoraria from Bristol‐Myers Squibb, UCB, Sun Pharma, Sanofi, Novartis, Beiersdorf AG, doctorderma Online Hautarzt and Melatech ApS, outside the submitted work; J.K. has received travel support from SUN Pharma and Pierre Fabre, outside the submitted work. J.U. is on the advisory board or has received honoraria and travel support from Amgen, Bristol Myers Squibb, GSK, Immunocore, LeoPharma, Merck Sharp and Dohme, Novartis, Pierre Fabre, Rheacell, Roche, and Sanofi outside the submitted work. S.A.W. received honoraria from Bristol Myers Squibb, Novartis, Pierre Fabre, and Sun Pharma outside the submitted work. G.G. has received honoraria from Almirall, BMS, SUN‐Pharma, Regeneron, Pfizer, Immunocore, and JanssenCilag, outside the submitted work. G.G. has received travel expenses from MSD, Pierre Fabre, SkylineDx, and SUN‐Pharma, outside the submitted work. G.G. has received grants from Regeneron and Delcath Systems, outside the submitted work. G.G. is a member of the advisory board of Regeneron. G.G. is a board member of SCOPE, unpaid. C.G. has received honoraria by Almirall, Biofrontera, BMS, Delcath, Immunocore, Janssen, Medscape, MSD, NAOS, Novartis, Onkowissen, Pierre Fabre, Regeneron, Sanofi, SUN Pharma, Sysmex. C.G. has received travel expenses by BMS, Pierre‐Fabre, SUN Pharma. C.G. has been a member of the advisory board of Almirall, Beiersdorf, BioNTech, BMS, Delcath, ImCheck, Immunocore, Immatics, Iovance, Moderna, MSD, Novartis, Pierre Fabre, Regeneron, Sanofi, SkylineDX, SUN Pharma, Sysmex. C.G. is a board member of the DeCOG (ADO), EADO, and MWS, unpaid; C.G. is a board member of the Hiege Stiftung, and the Roggenbuck‐Stiftung, unpaid. C.G. is co‐founder of Dermagnostix and Dermagnostix R&D. The other authors declare no conflicts of interest.

## Supporting information


**Table S1:** Cohort characteristics: completely resected melanoma.
**Table S2:** Cohort characteristics: advanced, metastatic melanoma.
**Table S3:** Univariate analysis of variables included in the Khorana risk score for venous thromboembolism in cancer patients.

## Data Availability

The data that support the findings of this study are available from the corresponding author upon reasonable request.
