# Supplementary material for: The Cancer Immunotherapy Thromboembolism Assessment: A Novel Score for Predicting Thromboembolic Events in Melanoma Patients Treated With Immune Checkpoint Inhibition
Source: Int J Cancer. 2026 Apr 27;159(5):1337–44. doi: 10.1002/ijc.70523 (PMC13340947; doi:10.1002/ijc.70523)
Supplement: Supplementary file 1 — Table S1: Cohort characteristics: completely resected melanoma. Table S2: Cohort characteristics: advanced, metastatic melanoma. Table S3: Univariate analysis of variables included in the Khorana risk score for venous thromboembolism in cancer patients. [file IJC-159-1337-s001.pdf]

The Cancer Immunotherapy Thromboembolism Assessment: A novel score for predicting thromboembolic events in melanoma patients treated with immune checkpoint inhibition

Tim Zell, Julian Kött, Noah Zimmermann, Greta Ancker, Alexander T. Bauer, Daniel J. Smit, Glenn Geidel, Julian C. Gerwers, Thomas Renné, Sebastian A. Wohlfeil, Jochen Utikal, Stefan W. Schneider, Christoffer Gebhardt

Table of Contents

Supplemental Table 1 Page 2

Supplemental Table 2 Page 3

Supplemental Table 3 Page 4

Supplemental Table 1

Cohort characteristics: completely resected melanoma

Variable	no.	(%)
**Age group**		
≥ 65	79	51.97
< 65	73	48.03
**Sex**		
Male	86	56.58
Female	66	43.42
**ECOG**		
0	136	89.47
≥ 1	16	10.53
**AJCC Stage**		
III	142	93.42
IV	10	6.58
**Measurable disease**		
No/post full resection	152	100.00
Yes	0	0.00

Supplemental Table 2

Cohort characteristics: advanced, metastatic melanoma

category	no.	(%)
**Age**		
≥ 65	117	56.80
< 65	89	43.20
**Sex**		
Male	128	62.14
Female	78	37.86
**ECOG Performance status**		
0	146	70.87
≥ 1	60	29.13
**AJCC Stage**		
III	41	19.90
IV	165	80.10
**Measurable disease**		
No/post full resection	0	0.00
Yes	206	100.00

Supplemental Table 3

Univariate analysis of variables included in the Khorana risk score for Venous Thromboembolism in Cancer Patients

Variable	n in group with TEE (%)	n in group without TEE (%)	Odds ratio	p-value
Platelet count ≥350x10⁹/L	62 (18.6%)	5 (20.8%)	1.1545	0.783
Leukocyte count >11x10⁹/L	18 (5.4%)	3 (12.5%)	2.508	0.152
Hemoglobin level <10 g/dL or using RBC growth factors	11 (3.3%)	1 (4.2%)	1.2767	0.818
BMI ≥35 kg/m²	21 (6.3%)	1 (4.2%)	0.648	0.676
